# Gastrointestinal Endoscopy Performed by Gastroenterologists: Opportunistic Screening Strategy for Newly Diagnosed Head and Neck Cancers

**DOI:** 10.3389/fonc.2022.793318

**Published:** 2022-05-27

**Authors:** Chih-Wei Yang, Yueng-Hsiang Chu, Hsin-Chien Chen, Wei-Chen Huang, Peng-Jen Chen, Wei-Kuo Chang

**Affiliations:** ^1^Division of Gastroenterology, Department of Internal Medicine, Tri-Service General Hospital, National Defense Medical Center, Taipei, Taiwan; ^2^Department of Otolaryngology-Head and Neck Surgery, Tri-Service General Hospital, National Defense Medical Center, Taipei, Taiwan

**Keywords:** upper gastrointestinal endoscopy, endoscopy, screening, cancer, head and neck cancer

## Abstract

**Aim:**

Approximately 66% of head and neck cancers are diagnosed at an advanced stage. This prospective study aimed to detect newly diagnosed head and neck cancers using regular upper gastrointestinal (UGI) endoscopy with oral-pharynx-larynx examination.

**Methods:**

A total of 2,849 patients underwent UGI endoscopy with an additional oral-pharynx-larynx examination. Patients aged < 20 years, those who were pregnant, had a history of head and neck cancers, were undergoing emergency endoscopy, and had a poor laryngopharyngeal view were excluded. The symptoms, incidence, location, pathology, and stage of malignant neoplasms were investigated.

**Results:**

A total of 2,720 patients were enrolled. Endoscopically observable 23 abnormal findings (0.85%) included 18 (0.66%) benign lesions and 5 (0.18%) newly diagnosed malignant neoplasms. Notably, 4 (80%) of 5 patients with malignant neoplasms were diagnosed at an early stage (Stage 0, I, and II).

**Conclusions:**

UGI endoscopy with oral-pharynx-larynx examination can achieve opportunistic head neck cancer screening and is recommended for every patient in endoscopy units.

## Introduction

Head and neck cancer is the sixth most common cancer worldwide (1). An estimated 53,000 new cases of head and neck cancer were reported in the United States in 2019 (2). Approximately 66% of head and cancer patients are diagnosed at an advanced stage and have a poor performance status (3). Gastrointestinal (GI) community studies reported that newly diagnosed head and neck cancer, ranging from 67% to 100% at an early curable stage, were incidentally detected with regular upper gastrointestinal (UGI) endoscopy (4–6).

Approximately 6 million upper endoscopies were performed in the United States in 2013 (7). The newly diagnosed cancer detection rate, ranging from 0.08% to 0.18%, was reported during regular UGI endoscopy (4–6). Assuming a newly diagnosed head and neck cancer detection rate of 0.1% for all endoscopies, regular upper endoscopy may provide an excellent opportunity to detect as many as 6,000 new potentially curable head and neck cancers each year.

Most national cancer screening programs are well organized and selective and target the population who is at the highest risk (8). The Taiwan Health Promotion Administration (HPA) provides national oral cancer screening for head and neck cancers (9). The HPA has commissioned the Taiwan Dental Association and the Taiwan Head and Neck Society to provide training on oral mucosa tests to dentists and ear, nose, and throat (ENT) doctors. The HPA has also authorized local governments to conduct oral mucus educational training for non-dental and non-ENT doctors once a year. A total of 412 non-dental and non-ENT doctors underwent this training in 2016. The HPA has collaborated with local health centers to hold practical training events at medical institutions conducting oral cancer testing and helped the trained doctors to perform opportunity cancer screening during daily practice (9, 10). The HPA regularly organizes a cancer screening education training program for non-dental and non-ENT physicians. This workshop was tailored toward GI physicians who provide care for patients at the endoscopy units and was designed to help them acquire the necessary knowledge and skill to promote head and neck cancer screening in the endoscopy units.

The oral cavity, pharynx, and larynx are located at the entrance to the esophagus and must be passed through during UGI endoscopy. GI endoscopists have performed opportunistic endoscopic screening at no additional cost; this was found effective on a large number of patients and offered both physicians and patients an opportunity for early detection of cancers (8, 11). However, the oral cavity, pharynx, and larynx are generally considered to be a field of otolaryngology. Gastroenterologists may be unfamiliar with the oral-pharynx-larynx examination. It is often impractical to ask patients to move their tongue upward and laterally to obtain a clear view of the oral cavity during the endoscopic examination. Endoscopic movements may cause the scope to touch the pharyngeal walls, trigger the coughing and gag reflex, and result in a poor laryngopharyngeal view. Several methods have been developed to overcome the challenges of UGI endoscopy with an additional oral-pharynx-larynx examination (6, 12–20).

UGI endoscopy with an additional oral-pharynx-larynx examination requires minimal additional time and is well tolerated by patients (6, 12, 14, 18, 19, 21). This prospective multidisciplinary study aimed to detect newly diagnosed head and neck cancers, by using regular UGI endoscopy with oral-pharynx-larynx examination.

## Materials and Methods

### Study Design

Patients undergoing regular UGI endoscopy at Tri-Service General Hospital, Taiwan, from December 2015 to December 2019 were included in the study. Patients aged < 20 years and those who were pregnant, undergoing emergency endoscopy, and had a poor laryngopharyngeal view were excluded. Before commencing the study, endoscopists were trained to perform UGI endoscopy with oral-pharynx-larynx examination, recognize the most common pathological findings, and summarize the possible pathologies. Demographic characteristics included sex, age, and presenting symptoms. Habits of cigarette smoking, alcohol drinking, and betel nut chewing were recorded. The present study was approved by the Institutional Review Board of the Tri-Service General Hospital, Taiwan (TSGHIRB 2-108-05-136). All methods were performed in accordance with the relevant guidelines and regulations. Patients were fully informed of the purpose of the study and they provided signed informed consent.

### Endoscopy Instruments

The UGI endoscopic examinations were performed using narrow-band imaging (EVIS LUCERA ELITE CVL-290; Olympus Optical Co Ltd, Tokyo, Japan) fitted with an endoscope (GIF-H260, GIF-H260Z, GIF-Q260, GIF-Q260Z, GIF-H290, GIF-HQ290, and GIF-H290Z; Olympus). Endoscopy with oral-pharynx-larynx examination was performed using a digital video recorder (HVO-550MD; Sony, Tokyo, Japan) (12, 15, 22).

### UGI Endoscopy With Oral-Pharynx-Larynx Examination

Patients were asked to fast for at least 4 hours (6, 12, 18, 23–25). Premedication varied according to the preference of the individual endoscopist, but consisted mostly of topical anesthesia, sometimes in combination with intravenous midazolam. Patients were placed in the left lateral decubitus position. The distal end of the endoscope was held approximately 30 cm away from the tip. The tip of the endoscope was inserted through a mouth-piece. With advancement of the endoscope along the midline of the palate, the uvula could be visualized over the base of the tongue. After the patient takes a deep breath, the epiglottis moves upward and forward, expanding and opening-up the larynx and vocal cord, which then provides a clear laryngopharyngeal view. The endoscope was rotated slightly, passed through the uvula, and gently advanced with anterior flexion to visualize the pyriform sinus, laryngeal vestibule, vocal cords, and upper part of the trachea. The vocal cords were observed at rest and during phonation of the word “e”. The pyriform sinus was inspected with minimal lateral deflection. Patients were monitored for their heart rate, electrocardiography findings, and oxygen saturation.

### Pathological and Diagnosis Confirmation

Endoscopically observable abnormal findings such as cysts, polyps, ulcers, leukoplakia, and telangiectasia in the oral-pharynx-larynx area were carefully investigated. Patients with endoscopically observable abnormal lesions were directly referred to an ENT specialist or oral surgeon for later review, where they underwent the standard method for examination and/or pathological confirmation. Premalignant or malignant neoplasms were histologically confirmed according to the World Health Organization criteria (26). The 8th edition of the American Joint Committee on Cancer (AJCC) was used for tumor staging (27). The treatment planning was conducted through tumor board review, which is composed of expert opinions from gastroenterologists, ENT specialists, radio-oncologists, and surgical, dental, and medical oncologists.

### Statistical Analysis

The data were analyzed using Excel 2010. Data are presented as means ± standard deviation for continuous variables with normal distribution and percentages (%) for categorical variables.

## Results

### Patient Characteristics

The characteristics of the 23 patients with endoscopically observable abnormal findings are shown in **Table 1**. The patients included 18 men and 5 women, aged 65.5 ± 12.4 years. Some patients had a habit of cigarette smoking (n = 17), alcohol consumption (n = 16), and betel nut chewing (n = 9). The presenting symptoms included globus sensation (n = 9), epigastric pain (n = 10), dysphagia (n = 9), GI bleeding (n = 2), acid regurgitation (n = 4), and body weight loss (n = 4).

**Table 1:** Characteristics of 23 patients with abnormal endoscopic findings.

Variable	Patients (n = 23)
Male/Female	18/5
Age (years), mean (SD)	65.5 ± 12.4
Smoking cigarettes, no. (%)	17 (74%)
Alcohol drinking, no. (%)	16 (70%)
Betel nut chewing, no. (%)	9 (39%)
Symptom, no. (%)	
Globus sensation	9 (39%)
Epigastric pain	10 (45%)
Dysphagia	9 (39%)
GI bleeding	2 (8%)
Acid regurgitation	4 (17%)
Body weight loss	4 (17%)

GI, gastrointestinal; no, number; SD, standard deviation.

### UGI Endoscopy With Oral-Pharynx-Larynx Examination

**Figure 1:**
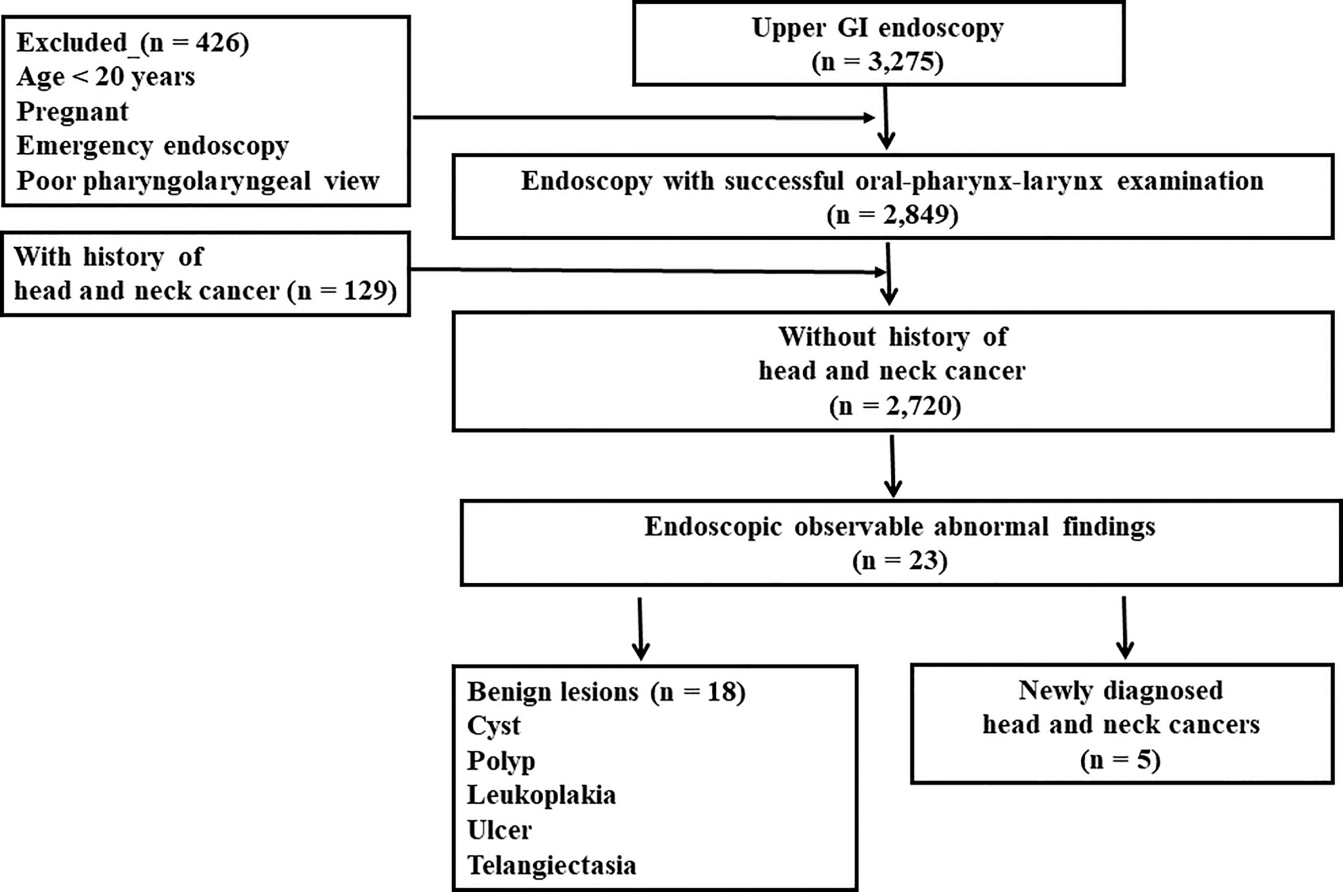
Flowchart of data processing. GI, gastrointestinal.

During the study period, UGI endoscopies were performed in 3,275 patients (**Figure 1**); 2,849 patients (87%) successfully completed UGI endoscopy with oral-pharynx-larynx examination. The added endoscopy time ranged from 17 to 60 seconds with a mean of 30 seconds.

### Endoscopically Observable Abnormal Findings

**Figure 2:**
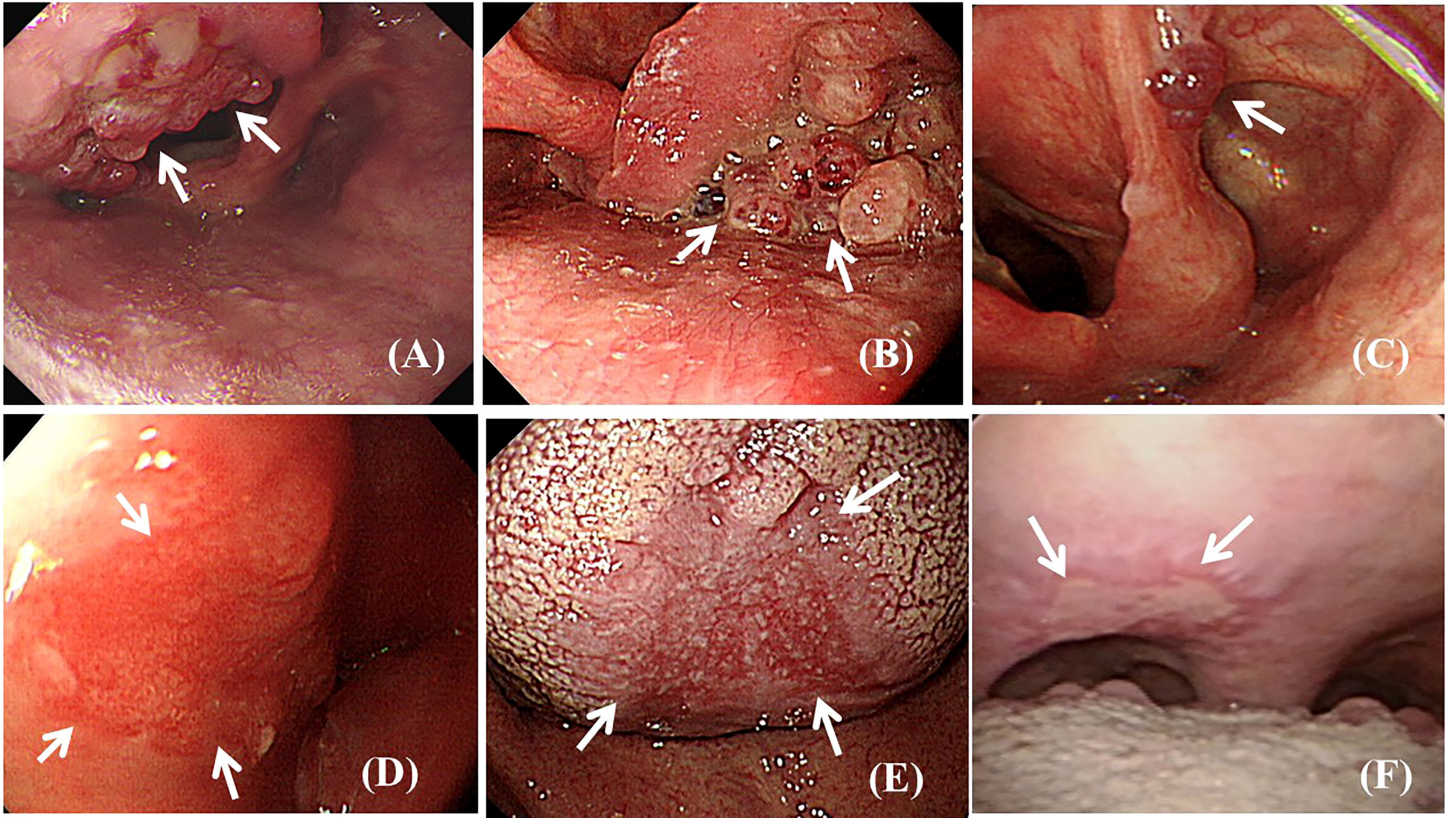
Endoscopic view of observable abnormal findings (arrows). **(A)** Polypoid squamous cell carcinoma lesion at the epiglottis. **(B)** Polypoid squamous cell carcinoma lesion at the left side piriform sinus. **(C)** Polypoid low-grade dysplasia lesion at the aryepiglottic fold. **(D)** Superficial flat squamous cell carcinoma lesion at the lateral wall of the hypopharynx. **(E)** Superficial flat squamous cell carcinoma lesion at the middle area of the dorsal tongue. **(F)** Superficial ulcerative low-grade dysplasia lesion at the soft palate.

Twenty-three (0.85%) endoscopically observable abnormal findings (**Figure 2**) were found, including 18 (0.66%) benign lesions (vocal cord cyst, n = 4; vocal cord polyp, n = 7; vocal cord leucoplakia, n = 1; oral ulcer, n = 3; and telangiectasia, n = 3) and 5 (0.18%) pathologically confirmed malignant neoplasms (squamous cell carcinoma, n = 5).

### Newly Diagnosed Malignant Neoplasms

Five newly diagnosed malignant neoplasms (0.18%) were found in 2,720 patients without a history of head and neck cancer (**Table 2**). All five patients had a habit of smoking cigarettes, alcohol drinking, or betel nut chewing (**Table 3**). The five newly diagnosed malignant neoplasms were located in the pharynx (n = 3), larynx (n = 1), and oral cavity (n = 1). Notably, 80% of malignant neoplasms (n = 4) were found at an early stage (AJCC stage 0, I, and II). Three patients (cases # 1, # 2, and # 3) were suitable for minimally invasive treatment, such as local surgery or endoscopic mucosal resection.

**Table 2:** Newly diagnosed head and neck cancers detected with regular UGI Endoscopy.

Study	Endoscopy with Oral-pharynx-larynx Examination			Newly diagnosed head and neck cancer			
	Total	Success		New cancer		Early stage^*^	
	(n)	(n)	(%)	(n)	(%)	(n)	(%)
Watanabe et al, 1996 (4)	N.R.	1,623	N.R.	3	0.18	2	67
Lehman et al, 1982 (5)	N.R.	1,120	N.R.	1	0.09	1	100
Mullhaupt et al, 2004 (6)	1,311	1,209	93	1	0.08	1	100
Current study	3,275	2,852	87	5	0.15	4	80

UGI, upper gastrointestinal; N.R, not recorded^*^; American Joint Committee on Cancer clinical stage 0, I, or II.

**Table 3:** Characteristics of five newly diagnosed head and neck cancers.

Case	Age	Sex	Symptom	Risk factor	Tumor location	Pathology	TNM system	AJCC stage	Treatment
1	56	M	Dysphagia	A, C	Pharynx	SCC	T_is_N_0_M_0_	0	Local surgery
2	62	M	Globus	A, C	Pharynx	SCC	T_1a_N_0_M_0_	I	EMR-C
3	64	M	Regurgitation	A, B, C	Oral cavity	SCC	T_1_N_0_M_0_	I	Local surgery
4	72	M	Dysphagia	A, C	Larynx	SCC	T_2_N_0_M_0_	II	CCRT
5	68	M	Dysphagia	A, B, C	Pharynx	SCC	T_3_N_2b_M_0_	IVA	CCRT, surgery

M, male; A, alcohol drinking; B, betel nut chewing; C, cigarette smoking; SCC, squamous cell carcinoma; EMR-C, endoscopic mucosal resection with a cap; CCRT, concurrent chemoradiotherapy; AJCC, American Joint Committee on Cancer; TNM, tumor, nodes, and metastases.

## Discussion

### Summary of New Highlights in This Manuscript

This is a large prospective study that reported observable abnormal findings in the oral cavity, pharynx, and larynx, using regular UGI endoscopy. Five newly diagnosed malignant neoplasms (0.18%) were observed in 2,720 patients without a history of head and neck cancer. Four (80%) of the newly diagnosed malignant neoplasms were at an early stage without obstructive symptoms, which made curative treatment possible. Upper GI endoscopy with oral-pharynx-larynx screening examination can provide several advantages regarding the necessary information for further therapeutic interventions. Our number of UGI endoscopies (n = 2,800) is similar to that seen in previous gastrointestinal (GI) community studies (n = 1120, n = 1623) that used UGI endoscopy with an additional oral-pharynx-larynx examination to perform opportunistic endoscopic head and neck cancer screening. Our results are similar to those of previous studies (**Table 2**), where the cancer detection rate ranged from 0.08% to 0.18%, with the proportion of early-stage cancers ranging from 67% to 100% (4–6).

### Majority of Newly Diagnosed Head and Neck Cancer Without Obstructive Symptoms

The Taiwan HPA provides national oral cancer screening for high-risk populations (9). Moreover, the HPA organizes an oral cancer screening training program for non-dental and non-ENT physicians. The HPA expects the trained GI physicians to monitor precancerous lesions and perform opportunistic oral cancer screening during daily practice (9, 10). The oral cavity, pharynx, and larynx are located at the entrance to the esophagus and must be passed through during UGI endoscopy. GI endoscopists should be aware of diseases of the oral-pharynx-larynx when performing UGI endoscopy. GI endoscopists have performed opportunistic endoscopic screening at no additional cost; this was found effective on a large number of patients and offered both physicians and patients an excellent opportunity for very early detection of cancers.

Symptom-directed, selective endoscopy is an efficient and cost-effective means to detect head and neck cancer (28). Head and neck cancer symptoms may include a lump in the neck, soreness in the mouth or throat that makes swallowing difficult, and a change or hoarseness in the voice. It is recommended that UGI endoscopy be conducted with an extra view of the blind spot of potential malignant neoplasms: the area underneath the tongue or in the space between the gum and cheeks. Unfortunately, majority of patients with laryngeal abnormalities (88%) did not report laryngeal symptoms, such as chronic hoarseness, throat clearing, or coughing, and would probably never have sought medical assistance. Thorough investigation of this area (oral-pharynx-larynx cavity) is considered mandatory to ensure provision of high quality endoscopic services. However, most GI endoscopists are unfamiliar with the oral-pharynx-larynx examination. Several gastrointestinal (GI) community studies using UGI endoscopy with an additional oral-pharynx-larynx examination have overcome the challenges of UGI endoscopy with an additional oral-pharynx-larynx examination. Newly diagnosed head and neck cancer, ranging from 67% to 100% at an early curable stage, were incidentally detected with regular UGI endoscopy (6, 12, 14, 15, 17, 19).

This study demonstrated that the majority of newly diagnosed malignant neoplasms were at an early stage without obstructive symptoms, which may be removed with minimally invasive treatment.

### UGI Endoscope for Oral-Larynx-Pharyngeal Screening

The commonly used UGI endoscope (GIF-H290, Olympus) is preferred to the ENT laryngoscope (ENF-VH, Olympus) for endoscopic oral-pharynx-larynx examination. GI endoscopes have a larger outer diameter (8.9 mm vs. 3.9 mm) than ENT endoscopes and are available with a suction channel without impairing optics, providing powerful magnification images (85 times optical magnification vs. no optical magnification). The longer working channel length (103 cm vs. 30 cm) ensures that tumor screening is not only focused on the oral cavity, pharynx, and larynx, but also is extended into the esophagus. Flexible UGI endoscopes can easily perform a delicate endoscope manipulation and show an abnormal lesion hidden in the piriform sinus, postcricoid area, or posterior pharyngeal wall.

Conventional GI endoscopists tend to pass the endoscope quickly through the throat, with the intention of minimizing patient discomfort and without trying to perform an oral-pharynx-larynx examination. There is no standard technique for UGI endoscopy with oral cavity, pharynx, or larynx examination. Several methods were developed in this study to overcome the challenges of oral-pharynx-larynx examination.

### GI Endoscopists Unfamiliar With the Oral-Pharynx-Larynx Examination

Successful UGI endoscopy for oral-pharynx-larynx examination requires adequate knowledge and procedural skills. However, current GI specialist training programs do not provide structured training in the ENT field. GI specialists are unfamiliar with UGI endoscopy when used for oral-pharynx-larynx examination. Therefore, there is a need to develop multidisciplinary teamwork and training programs for the implementation of UGI endoscopy with oral-pharynx-larynx examination.

GI specialists can never replace ENT specialists in the management of oral, pharyngeal, and laryngeal disorders. Oral cavity, pharynx, or larynx biopsies are not performed by GI endoscopists. A biopsy may cause pain because of a lack of local anesthesia and risk of bleeding (5, 14). After initial detection of an abnormal finding, patients should be directly referred to ENT specialists or oral surgeons for further diagnosis and treatment. Video recordings can be used to review the endoscopic examination later on a monitor, which can reduce the possibility of missed observations (15, 22, 29, 30). Video recordings can also be used for communication, teaching, research, and education. Connection of a video recording system is recommended for endoscopy with oral-pharynx-larynx examination (6, 12, 15, 19).

### Simplifying the Endoscopic Oral Examination

It was not practical to ask patients to move their tongue upward and laterally to obtain a clear view of the oral cavity during the endoscopic examination. It was especially difficult for patients who were critically ill, having neurological conditions, or under conscious sedation. To simplify the endoscopic oral examination, we did not routinely screen lesions hidden underneath the tongue or in the space between the gum and cheeks. Therefore, endoscopic oral examination can only have a view of the lips, palate, uvula, and dorsal surface of the tongue.

### Having a Clear Laryngopharyngeal View

When the scope passes the uvula, the epiglottis may guard and cover the opening of the larynx and vocal cords and present an anatomically closed field; therefore, the scope cannot provide a clear view of the laryngopharyngeal area. When the patient takes a deep breath, the epiglottis moves upward and forward; the larynx and vocal cord then expand and open up and provide a wide and clear laryngopharyngeal view.

*T*he accumulation and pooling of secretions might fill in the lowermost area of the right side of the pyriform sinus, flow into the laryngeal vestibule or vocal cords, and increase the risk of aspiration pneumonia. To minimize the risk of aspiration and ensure a clear endoscopic view, secretions in the pharyngolaryngeal region were suctioned before inserting the endoscope into the esophagus.

### Minimizing the Coughing and Gag Reflex

Topical pharyngeal sprays with anesthetic agents can be directed to the posterior pharyngeal wall to suppress coughing and gag reflex (22). A prospective study of 2,000 UGI endoscopic examinations without sedation and topical pharyngeal anesthesia (10% lidocaine spray) reported a safe, quick, and well-tolerated procedure (31). Topical pharyngeal anesthesia is not beneficial for conscious sedated patients (32).

Notably, a compassionate, relaxed, physician with a soothing bedside manner may be more effective than a topical pharyngeal spray (33). GI endoscopists may hold the distal end of the endoscope approximately 30 cm away from the tip (18). Therefore, when the scope passes from the uvula and pharynx into the upper esophagus, it is not necessary to change the hand-holding position, and it is much easier to make a delicate endoscope manipulation. Careful and slow endoscopic movements can avoid touching the pharyngeal walls. Creating an air or water infusion may irritate the throat or airway and trigger the coughing and gag reflex (6, 15, 22).

### Limitations

The oral cavity, pharynx, and larynx are generally considered to be a field of otolaryngology. Limited training in the field of otolaryngology and the use of conventional vs. high-resolution endoscopic equipment could be considered as limitations. UGI endoscopy may have a blind spot when potential malignant neoplasms are located underneath the tongue or in the space between the gum and cheeks. This single-center study was conducted in Taiwan; therefore, the findings may not be generalizable to other populations. Further larger-scale study is necessary to develop an optimal screening program for head and neck cancers across different populations.

## Conclusions

The UGI endoscope passes into the esophagus and with careful maneuvering and observation, GI endoscopists were able to extend observation into the oral cavity, pharynx, and larynx. UGI endoscopy with an additional oral-pharynx-larynx examination required minimal additional time, at no additional cost, and was well tolerated by patients. Most of the endoscopically observable malignant neoplasms were detected early enough for curative therapy. UGI endoscopy with oral-pharynx-larynx examination can achieve opportunistic head neck cancer screening and is recommended for every patient in endoscopy units.

## Data Availability Statement

The original contributions presented in the study are included in the article/supplementary material. Further inquiries can be directed to the corresponding author.

## Ethics Statement

The studies involving human participants were reviewed and approved by the Institutional Review Board of the Tri-Service General Hospital, Taiwan (TSGHIRB 2-108-05-136). The patients/participants provided their written informed consent to participate in this study.

## Author Contributions

C-WY contributed to conception, design, and analysis, and interpretation of data. Y-HC, and H-CC contributed to conception, design, analysis and interpretation of data. W-CH and P-JC contributed to the analysis plan and wrote the manuscript. W-KC contributed to the study design and supervised the study. All authors reviewed the manuscript. All authors contributed to the article and approved the submitted version.

## Funding

We are grateful for the financial support provided by the Ministry of National Defense-Medical Affairs Bureau, Tri-Service General Hospital (TSGH-C108-070), Taiwan for this study.

## Conflict of Interest

The authors declare that the research was conducted in the absence of any commercial or financial relationships that could be construed as a potential conflict of interest.

## Publisher’s Note

All claims expressed in this article are solely those of the authors and do not necessarily represent those of their affiliated organizations, or those of the publisher, the editors and the reviewers. Any product that may be evaluated in this article, or claim that may be made by its manufacturer, is not guaranteed or endorsed by the publisher.
